# Supplementary material for: High-resolution ultramicroscopy of the developing and adult nervous system in optically cleared Drosophila melanogaster
Source: Nat Commun. 2018 Nov 9;9:4731. doi: 10.1038/s41467-018-07192-z (PMC6226481; doi:10.1038/s41467-018-07192-z)
Supplement: Supplementary file 10 — Supplementary Information [file 41467_2018_7192_MOESM10_ESM.pdf]

# High-resolution ultramicroscopy of the developing and adult nervous system in optically cleared *Drosophila melanogaster*

Pende et al.

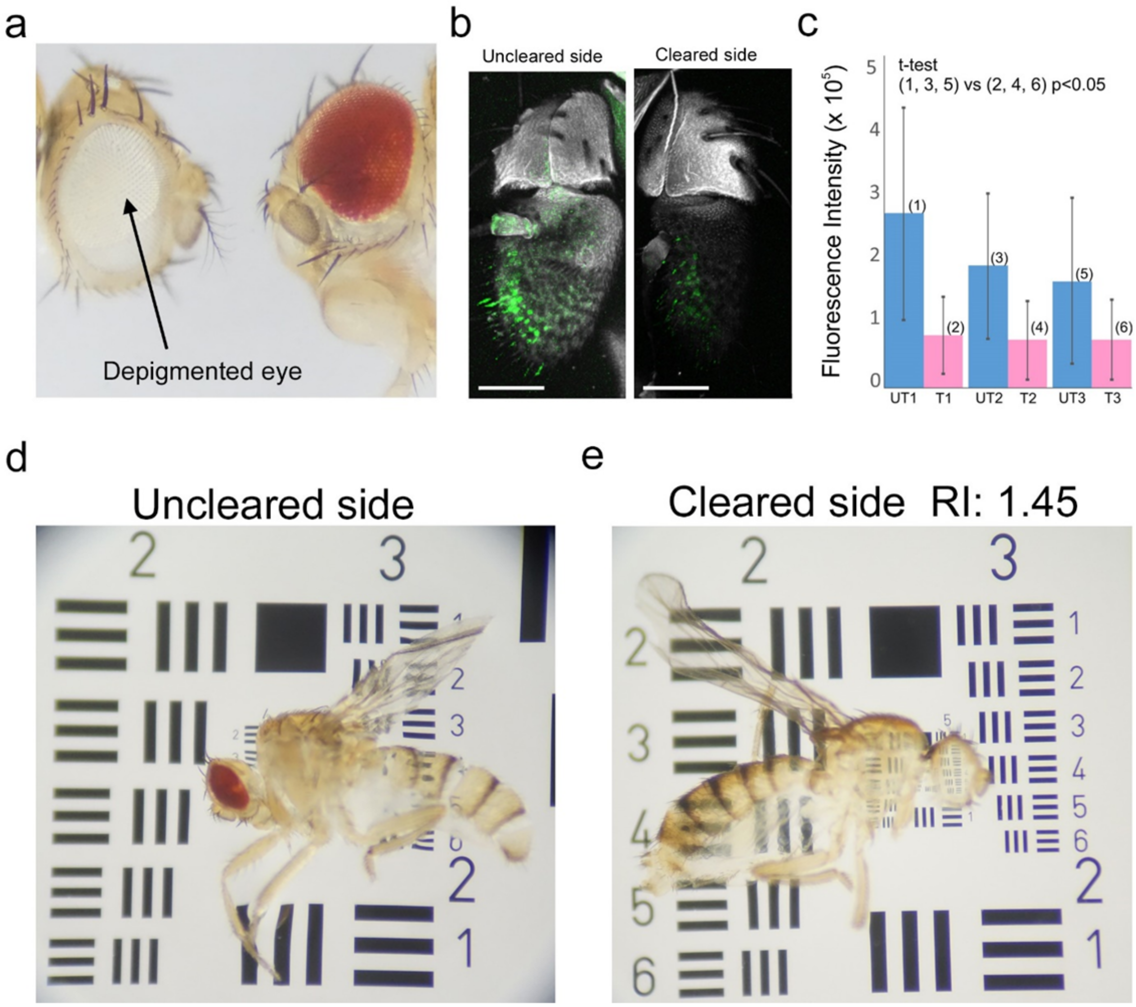


**Supplementary Figure 1** | Comparison of uncleared and FlyClear treated adult flies. **a**, **d** and **e** Incident light image showing comparison of uncleared and cleared sides of the same fly. **b** Confocal image of weak GFP signal in the maxillary palp of uncleared and cleared sides of the same fly. **a** Comparison of the depigmentation properties of *Solution-1*. *Solution-1* treated side shows complete depigmentation of the adult eye (image was taken in PBS). **b** Comparison of GFP signal in Uncleared and four days in *Solution-1* cleared sample. Both sides of the same fly were mounted on one slide in Vectashield.and recorded with the same laser intensity. **c** Diagram shows the results from three independent experiments comparing the intensities (± s.d.) of untreated (UT) and *Solution-1* treated (T) sides of flies (n=3). Significance was assigned with unpaired t-test ($p<0.05$). **d** Uncleared right side of adult fly **e** FlyClear processed cleared left side of the same fly. Images in **a**, **d** and **e** were acquired with a stereomicroscope with a 1x objective (Leica, Plan APO 1.0X, WD 61,5mm). Images in **b** were acquired with a 20x immersion objective (Leica, HCX PL APO CS, 0.7 NA, 260µm WD). Genotype: *; UAS-mCD8:GFP ; Or47b-Gal4*. Scale bars in **b** represent 50µm.

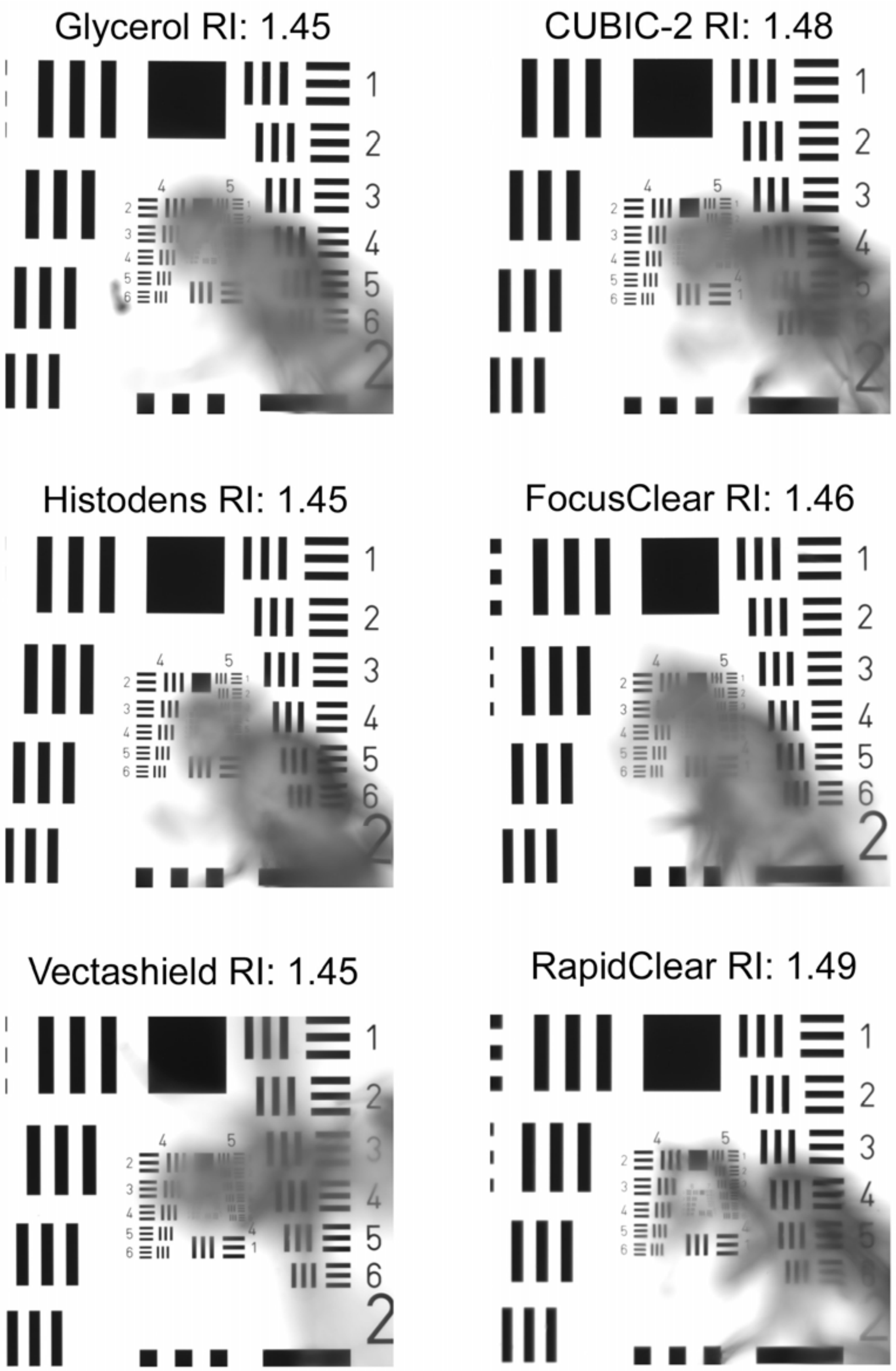


**Supplementary Figure 2** | Comparison of transparency achieved after treatment with *Solution-1* and commercial refractive index (RI) matching media. Wide-field image of optically cleared specimens placed on top of a USAF1951-chart.

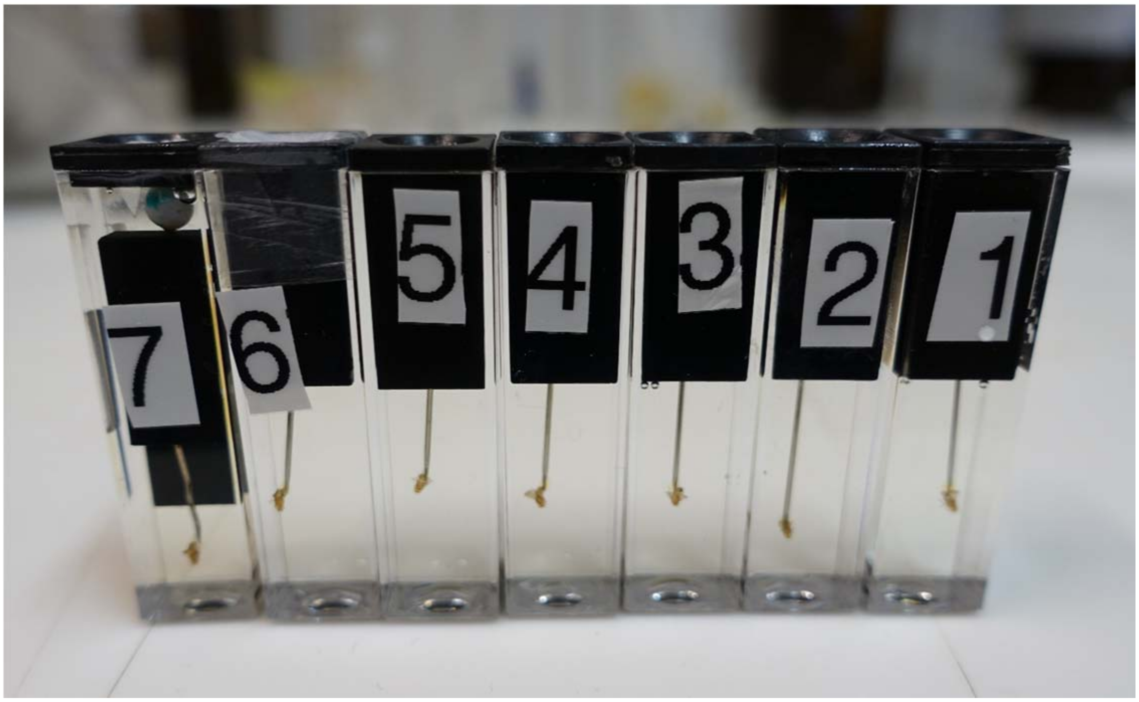


**Supplementary Figure 3** | Mounting of optically cleared flies for signal quantification. Optically cleared flies were mounted with a UV glue on a needle tip, immersed in a cuvette filled with *Solution-2,* and sealed for imaging and storage.

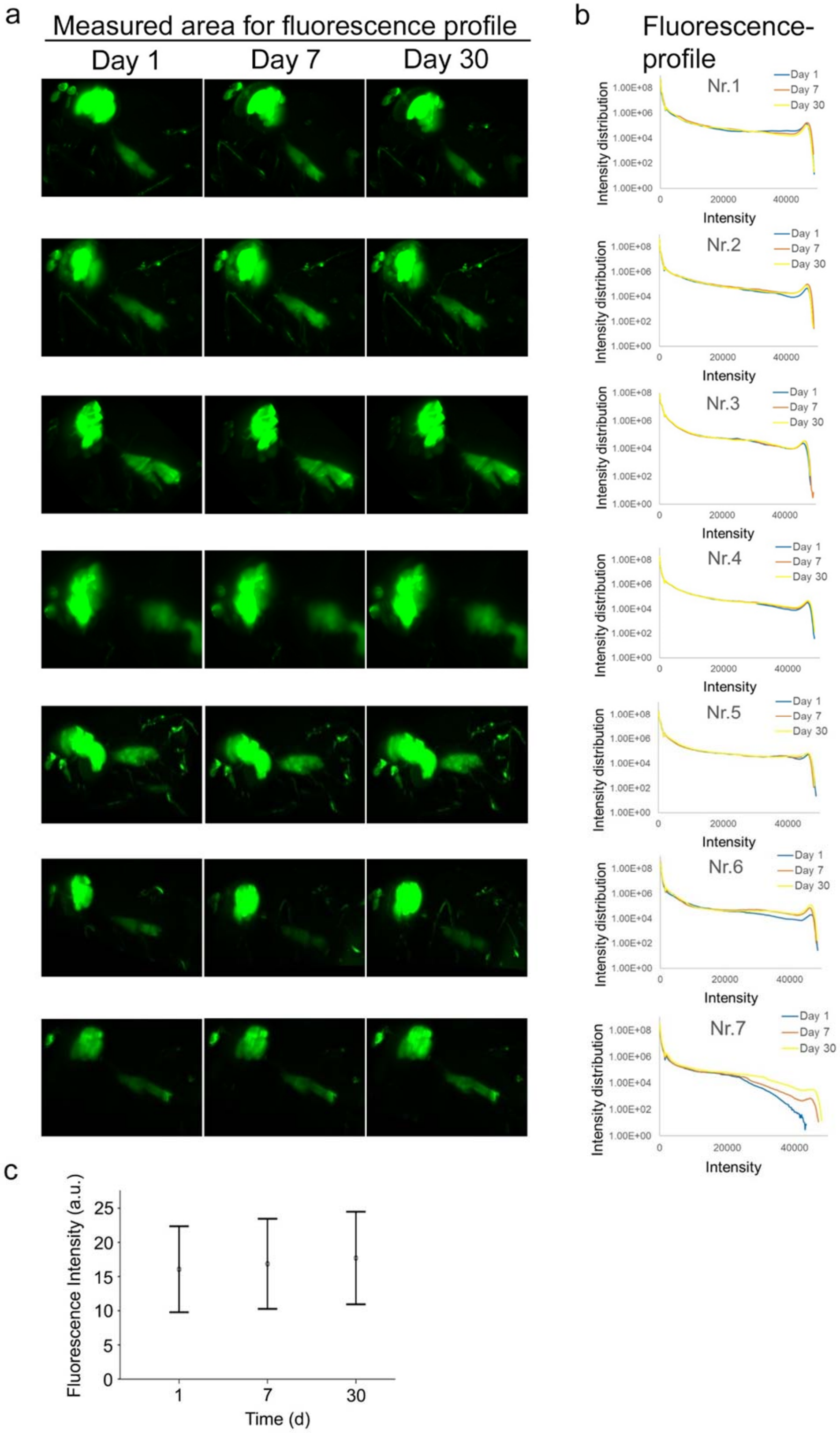
a
Measured area for fluorescence profile
Day 1
Day 7
Day 30
b
Fluorescence-profile
Nr.1
Nr.2
Nr.3
Nr.4
Nr.5
Nr.6
Nr.7
Day 1
Day 7
Day 30
Intensity distribution
1.00E+08
1.00E+06
1.00E+04
1.00E+02
1.00E+00
0
20000
40000
Intensity
c
Fluorescence Intensity (a.u.)
25
20
15
10
5
0
1
7
30
Time (d)

**Supplementary Figure 4** | Stability of the GFP signal in adult fly after FlyClear procedure. **a** Light-sheet stacks of 600 images show GFP signal in *Drosophila melanogaster* measured with the same settings in the same areas at three different time points (n=7). **b** Histogram data showing the comparison of fluorescence level profiles within these areas over time. **c** Dotplot representing the average ± s.d. of the intensity distribution from seven independent measurements after one day, one week, and one month. For measuring significance, p-values were assessed with one-way ANOVA ($P<0.911$). All images were acquired with a 4x Objective (Olympus, XLFluor4x/340, 0.28 NA, WD = 29,5mm) with custom-made correction of optics for a refractive index of 1.45 (WD after correction 10mm). Genotype: *dscam-Gal4/CyO; UAS-mCD8::GFP*

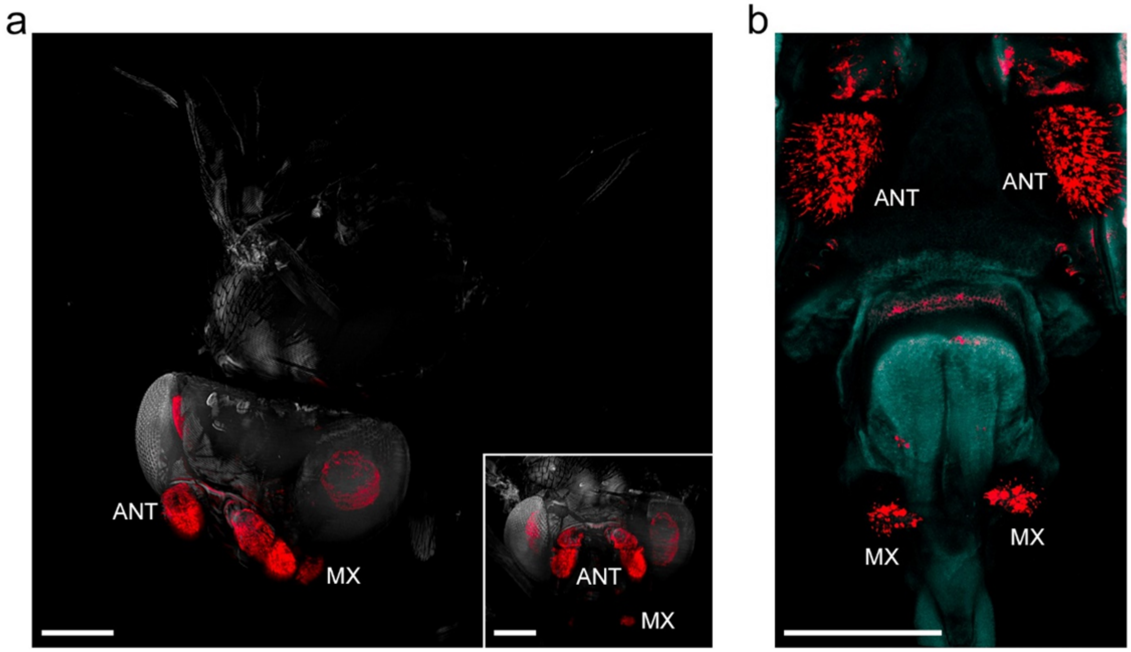


**Supplementary Figure 5** | Visualisation of mCherry labeled adult *Drosophila* chemosensory system using FlyClear with confocal- and optimised ultramicroscope. **a** Light-sheet images and **b** confocal images of mCherry expression. **a** Olfactory neurons in antenna (ANT) and maxillary palp (MX). Inset shows coronal view of fly head. **b** Higher magnification of ANT and MX. Images in **a** were acquired with 0.5x post-magnification in combination with a 10x water-immersion objective (Olympus, UMPlanFLN, 0.3 NA, WD = 3.5mm) with custom-made correction of optics for a refractive index of 1.45 (WD = 3.5mm after correction). Image in **b** was acquired with a 20x immersion objective (Leica, HCX PL APO CS, 0.7 NA, 260µm WD). Genotype: *;;R88E12-Gal4 UAS-mCherry*. Scale bars represent 200µm.

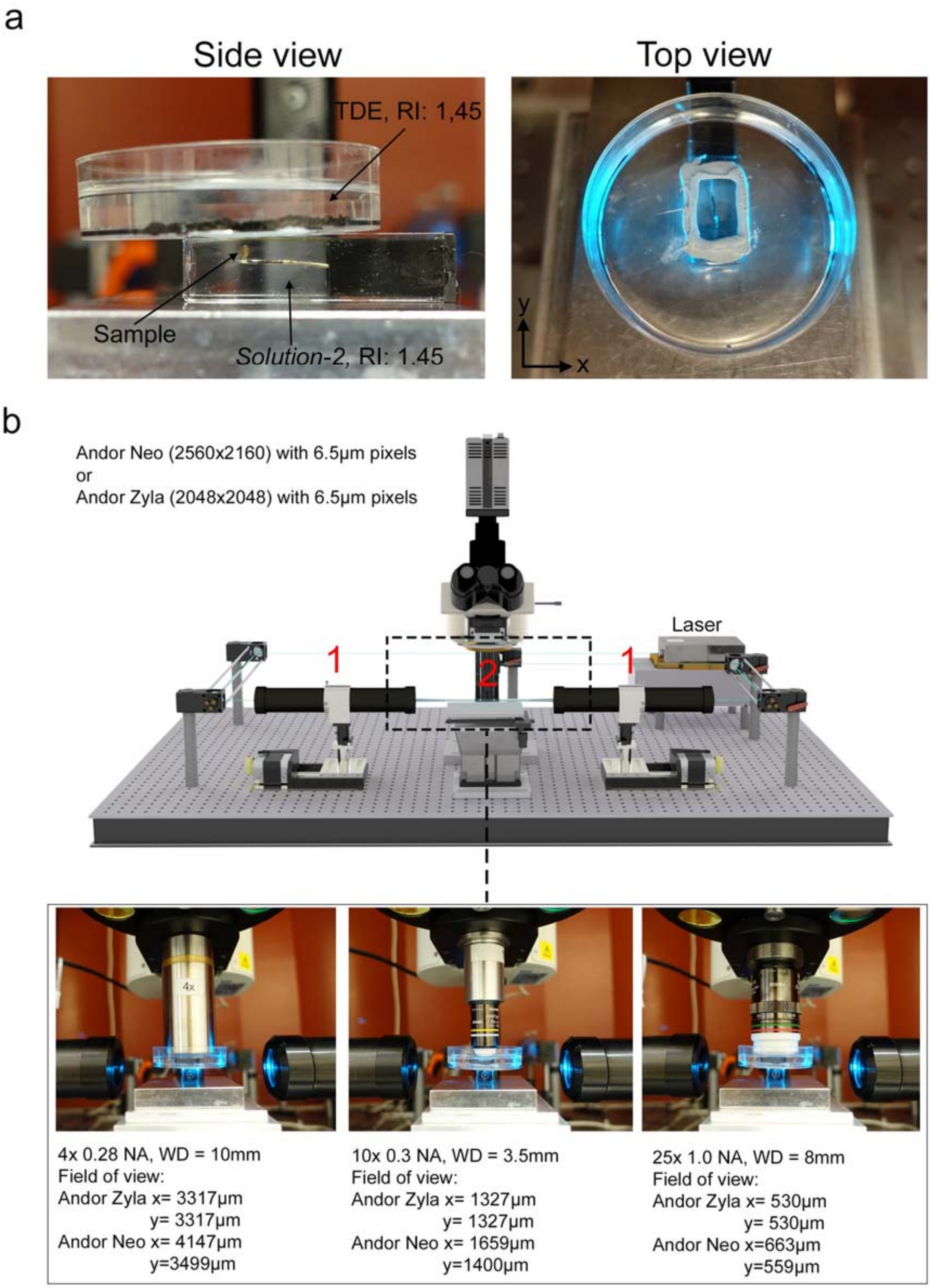
a
Side view
Top view
TDE, RI: 1,45
Sample
Solution-2, RI: 1.45
y
x
b
Andor Neo (2560x2160) with 6.5µm pixels
or
Andor Zyla (2048x2048) with 6.5µm pixels
Laser
1
2
1
4x
4x 0.28 NA, WD = 10mm
Field of view:
Andor Zyla x= 3317µm
y= 3317µm
Andor Neo x= 4147µm
y=3499µm
10x 0.3 NA, WD = 3.5mm
Field of view:
Andor Zyla x= 1327µm
y= 1327µm
Andor Neo x= 1659µm
y=1400µm
25x 1.0 NA, WD = 8mm
Field of view:
Andor Zyla x= 530µm
y= 530µm
Andor Neo x=663µm
y=559µm

**Supplementary Figure 6** | Ultramicroscope system and imaging chamber (a) Side and top view of the imaging chamber. The sample containing chamber is filled with *Solution-2* (RI: 1,45) the objective immersion chamber is filled with aqueous Thiodiethanol solution (RI: 1,45). (b) Scheme of the ultramicroscope system. (1) Light-sheet generator (illumination lens), (2) detection lenses. Boxed area indicates different detection lenses used for imaging. (From left to right) 4x Objective (Olympus, XLFluor4x/340, 0.28 NA, WD = 29,5mm) with custom-made correction of optics for a refractive index of 1.45 (WD after correction 10mm), 10x water-immersion objective (Olympus, UMPlanFLN, 0.3 NA, WD = 3.5mm) with custom-made correction of optics for a refractive index of 1.45 (WD = 3.5mm after correction), 25x objective (Olympus, XLPlanN, 1.0 NA, WD = 8mm)

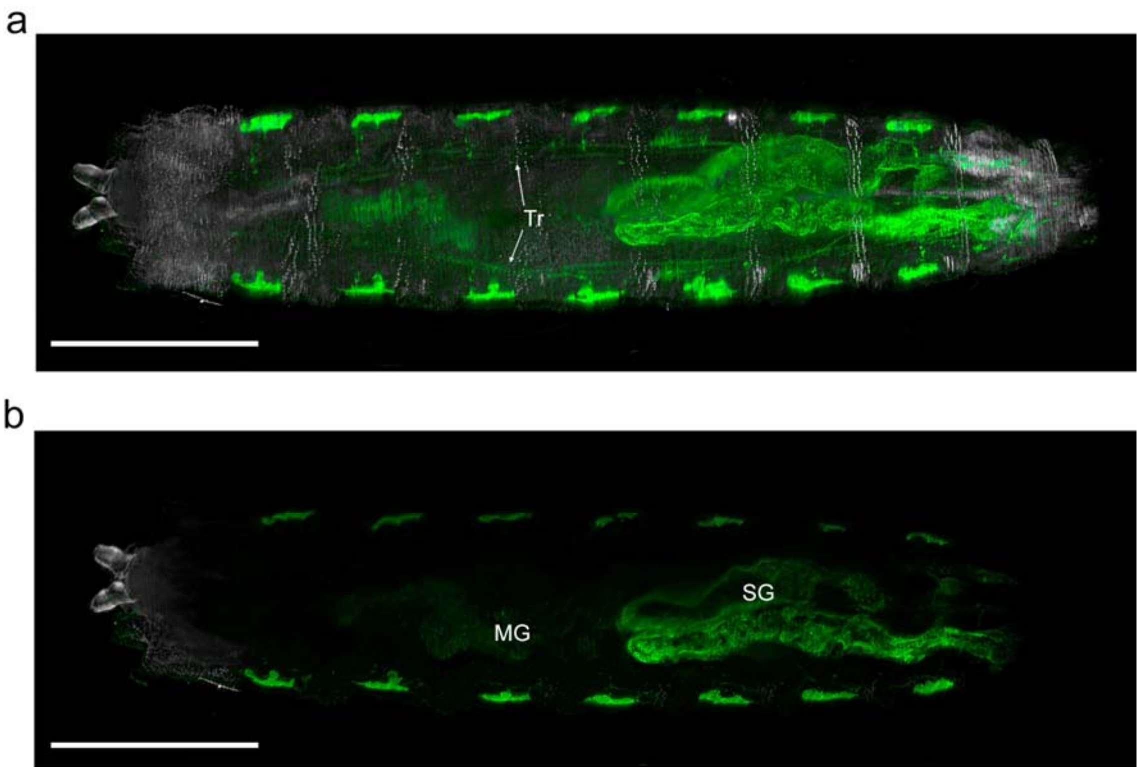


**Supplementary Figure 7** | Imaging of FlyClear treated second instar larva using optimised ultramicroscope system. **a, b** Light-sheet images of GFP expression in larval organs. **a** Imaging of whole larva showing the trachea (Tr). **b** Dorsal view of a clipping plane in undisected larva showing mid gut (MG) and salivary glands (SG). All images were acquired with a 10x water-immersion objective (Olympus, UMPlanFLN, 0.3 NA, WD = 3.5mm) with custom-made correction of optics for a refractive index of 1.45 (WD = 3.5mm after correction). Genotype: *Peb-Gal4 UAS-mCD8::GFP.* Scale bars represent 500µm.

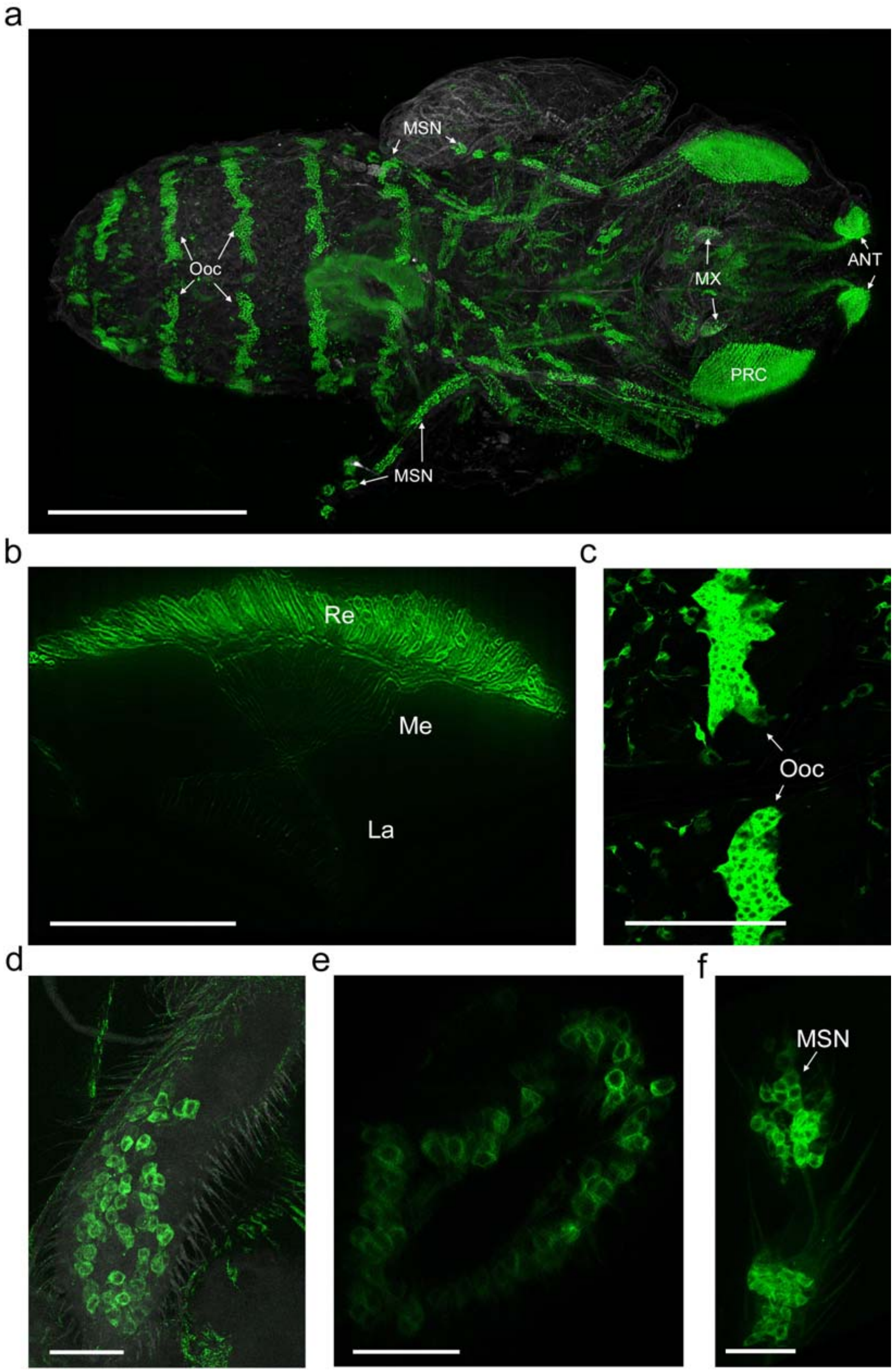
a
MSN
Ooc
MX
ANT
PRC
MSN
b
Re
Me
La
c
Ooc
d
e
f
MSN

**Supplementary Figure 8 |** Imaging of reproductive, visual and sensory system of an intact pupa using confocal- and optimised aspheric ultramicroscope. **a**, **b** Light-sheet images and **c**-**f** confocal images of GFP expression. **a** Ventral view of Pebbled expression in whole pupa. Arrows indicate oocytes (Ooc) in abdomen, mechanosensory neurons (MSN) in the lags, photoreceptor cells (PCR) in the eyes and olfactory neurons in the maxillary palps (MX) and antenna (ANT). **b** Photoreceptor projecting from the retina (Re) through the lamina (La) into the medulla (Me). **c** oocytes, olfactory neurons in **d** maxillary palp and **e** antenna and **f** mechansensory neurons in leg tip. Image **a** was acquired with 0.5x post-magnification in combination with a 10x water-immersion objective (Olympus, UMPlanFLN, 0.3 NA, WD = 3.5mm) with custom-made correction of optics for a refractive index of 1.45 (WD = 3.5mm after correction). Image in **b** was acquired with a 2x post-magnification in combination with a 25x objective (Olympus, XLPlanN, 1.0 NA, WD = 8mm). Confocal images in **c**, **d** were acquired with a 40x Oil-immersion objective (Leica, HCX OL APO CS, 1.25 NA, 100µm WD) and in **e**, **f** with a 63x Glycerol-immersion objective (Leica, HC PL APO, 1.3 NA, WD 280µm). Genotype: *Peb-Gal4 UAS-mCD8::GFP.* Scale bars represent 500µm in **a**, 100µm in **b**, **c** and 25µm in **d**-**f**.

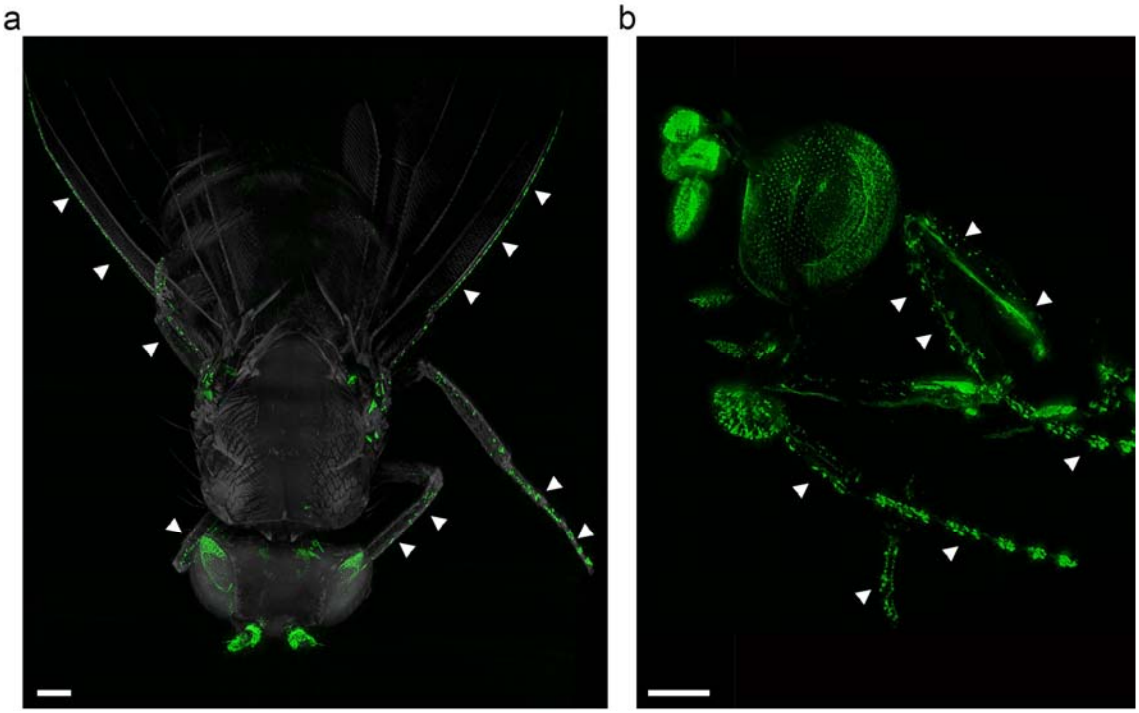


**Supplementary Figure 9** | Distribution of mechanosensory neurons in legs and wings visualised using optimised aspheric ultramicroscope system. **a, b** Light-sheet images of GFP expression. **a** Dorsal view of sensory neurons in legs and wings marked with arrowheads. **b** Lateral view of sensory neurons in legs and wings marked with arrowheads. Images in **a** was acquired with 0.5x post-magnification in combination with a 10x water- immersion objective (Olympus, UMPlanFLN, 0.3 NA, WD = 3.5mm) with custom made correction of optics for a refractive index of 1.45 (WD after correction 3.5mm). Image in **b** was acquired with 2x post-magnification in combination with a 4x Objective (Olympus, XLFluor4x/340, 0.28 NA, WD = 29,5mm) with custom-made correction of optics for a refractive index of 1.45 (WD after correction 10mm). In **a** a post magnification of 0.5x was used. Genotype: *Peb-Gal4 UAS-mCD8::GFP.* Scale bars represent 200µm.

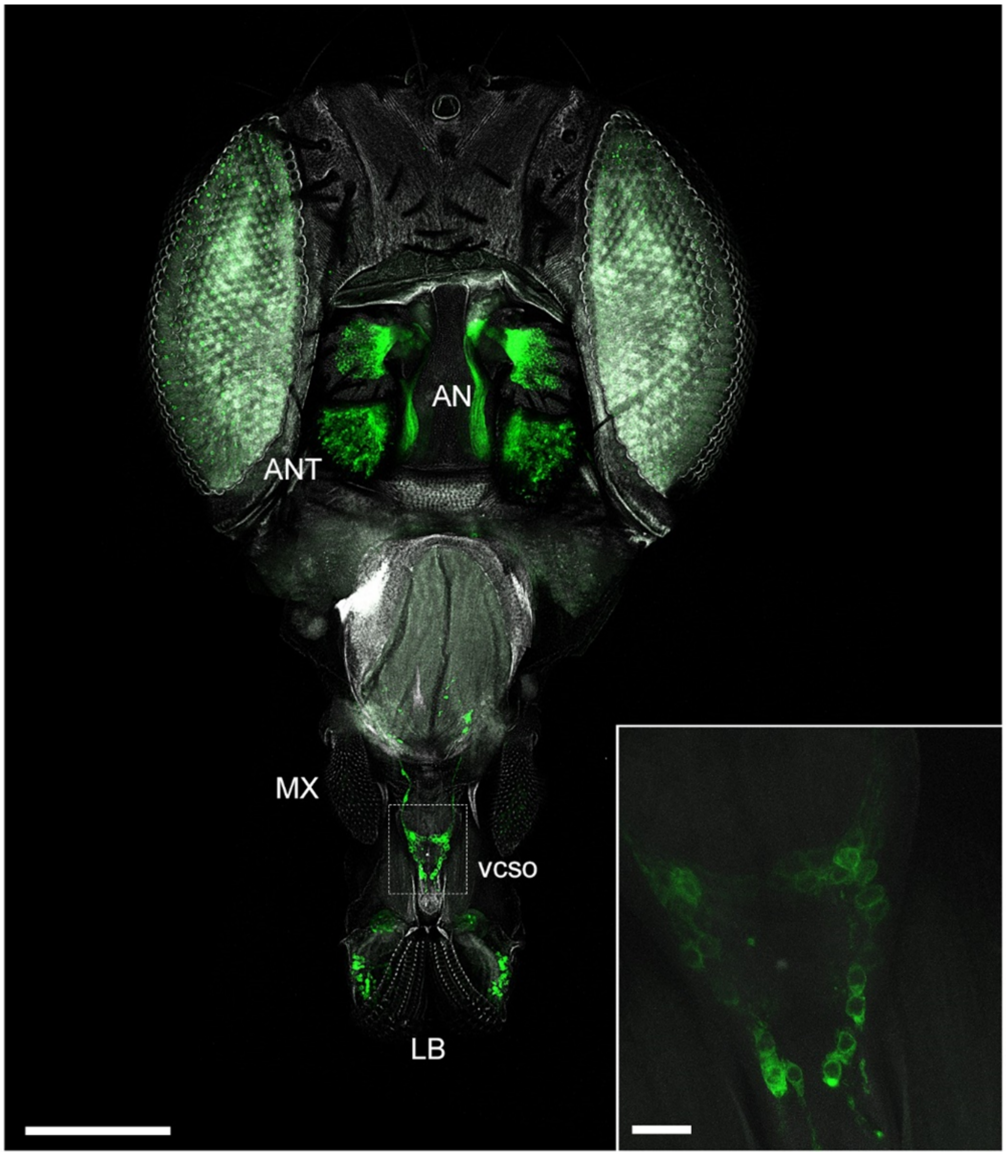


**Supplementary Figure 10** | Visualisation of the connections in adult fly olfactory receptor system using confocal microscopy. Coronal view of olfactory receptor neurons in antenna (ANT), maxillary palp (MX), labellum (LB), and part of the antennal nerve (AN) projecting from the ANT. Inset shows a higher magnification image of ventral cibarial sense organ (vcso). Images were acquired with a 20x immersion objective (Leica, HCX PL APO CS, 0.7 NA, 260µm WD). Genotype: *Peb-Gal4 UAS-mCD8::GFP.* Scale bars represent 200µm and 10µm in the main figure and inset respectively.

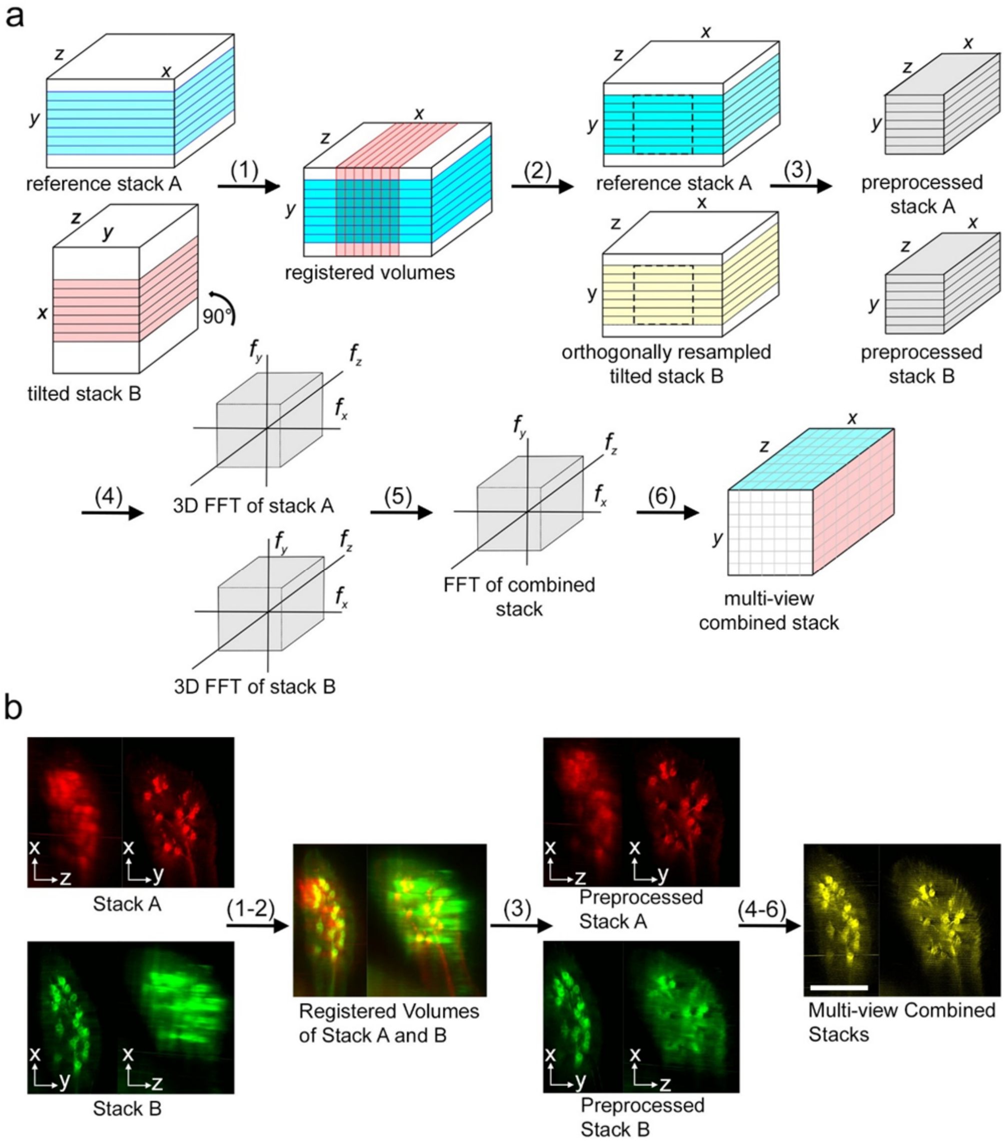


**Supplementary Figure 11** | Principle of multi-view combining of ultramicroscope-recordings from 90°tilted directions. **a** Scheme of the workflow of multi-view combining. **b** GFP-labelled neurons in maxillary palp processed with multi-view combining. **a, b** (1) Two image stacks, A (reference stack) and B (tilted stack) were recorded from orthogonally tilted directions and spatially registered using Amira software. (2) After registration, stack B was virtually resliced to generate computed section planes that are coplanar with respect to stack A. (3) Non-overlapping regions were removed from stacks A and B using a binary masking operation. The remaining parts were scaled to approximately the same average brightness by scaling to the 95% percentile. (4) A 3D Fast Fourier Transformation was applied to both stacks and the calculated magnitudes and phases were recombined according to eq. *(3)* and *(4)* in the methods section. (5) The multi-view combined stack comprising the sharper components of A and B was obtained by applying the inverse Fourier transformation.

(6) Finished multi-view combined stack. Scale bar represents 50µm and is representative for all images in **b**.

**Supplementary Table 1 |** Resolution of the USAF1951-chart in µm

| | Group Number | | | | | | |
|---|---|---|---|---|---|---|---|
| Element | 1 | 2 | 3 | 4 | 5 | 6 | 7 |
| 1 | 250 | 125 | 62.5 | 31.25 | 15.63 | 7.81 | 3.91 |
| 2 | 222.72 | 111.36 | 55.68 | 27.84 | 13.92 | 6.96 | 3.48 |
| 3 | 198.43 | 99.21 | 49.61 | 24.8 | 12.4 | 6.2 | 3.1 |
| 4 | 176.78 | 88.39 | 44.19 | 22.1 | 11.05 | 5.52 | 2.76 |
| 5 | 157.49 | 78.75 | 39.37 | 19.69 | 9.84 | 4.92 | 2.46 |
| 6 | 140.31 | 70.15 | 35.08 | 17.54 | 8.77 | 4.38 | 2.19 |

**Supplementary Table 2 |** Imaging specifications

| Figure | Animal | System | Objective | Postmagnification | Image pixel size | z-step | Number of channels | Imaging depth | Number of merged stacks | Data volume | Imaging Time |
|---|---|---|---|---|---|---|---|---|---|---|---|
| 3a | Prepupa | UM | corr. 10x RI: 1.45 | | 0.651µm x 0.651µm | 0.651µm | 2 | 936µm | 2 | 19GB | 2h |
| 3b | Prepupa | UM | corr. 10x RI: 1.45 | | 0.651µm x 0.651µm | 0.651µm | 2 | Clipped plane of 3a | | n.a | n.a |
| 3c | Prepupa | UM | corr. 10x RI: 1.45 | | 0.651µm x 0.651µm | 0.651µm | 2 | Clipped plane of 3a | 2 | n.a | n.a |
| 3d | Prepupa | Confocal | Leica 20x Gly | | 0.757µm x 0.757µm | 2.01µm | 2 | 247µm | | 190MB | 11min |
| 3e | Prepupa | Confocal | Leica 20x Gly | | 0.757µm x 0.757µm | 1.3µm | 2 | 119µm | | 260MB | 8min |
| | | | | | | | | | | | |
| 4a | adult *Drosophila* | UM | corr. 10x RI: 1.45 | | 0.651µm x 0.651µm | 0.651µm | 2 | 563µm | | 4GB | 40min |
| 4b | adult *Drosophila* | UM | corr. 10x RI: 1.45 | | 0.651µm x 0.651µm | 0.651µm | 2 | 546µm | | 4GB | 40min |
| 4c | adult *Drosophila* | UM | Olympus 25x RI:1.45 | | 0.259µm x 0.259µm | 0.259µm | 2 | 226µm | | 12GB | 45min |
| 4d | adult *Drosophila* | UM | Olympus 25x RI:1.45 | | 0.259µm x 0.259µm | 0.259µm | 2 | 196µm | | 10GB | 40min |
| 4e | adult *Drosophila* | UM | corr. 10x RI: 1.45 | | 0.651µm x 0.651µm | 0.651µm | 2 | 459µm | | 4GB | 35min |
| 4f | adult *Drosophila* | UM | Olympus 25x RI:1.45 | | 0.259µm x 0.259µm | 0.259µm | 2 | 248µm | | 16GB | 45min |
| | | | | | | | | | | | |
| 5a | adult *Drosophila* | UM | corr. 10x RI: 1.45 | 2x | 0.324µm x 0.324µm | 1.539µm | 2 | 672µm | 2 | 4GB | 40min |
| 5b | adult *Drosophila* | UM | corr. 10x RI: 1.45 | 2x | 0.324µm x 0.324µm | 1.539µm | 1 | 672µm | 2 | 2GB | 20min |
| 5c | adult *Drosophila* | UM | corr. 10x RI: 1.45 | | 0.651µm x 0.651µm | 0.651µm | 2 | 601µm | | 4GB | 40min |
| 5d | adult *Drosophila* | UM | Olympus 25x RI:1.45 | 0.5x | 0.54µm x 0.54µm | 1.08µm | 2 | 844µm | | 7GB | 55min |
| | | | | | | | | | | | |
| 6c | adult *Drosophila* | UM | corr. 4x RI: 1.45 | 2x | 0.81µm x 0.81µm | 1.62µm | 2 | 1313µm | | 3GB | 30min |
| 6d | adult *Drosophila* | UM | corr. 4x RI: 1.45 | 2x | 0.81µm x 0.81µm | 1.62µm | 2 | 2198µm | 2 | 10GB | 55min |
| 6e | adult *Drosophila* | UM | corr. 4x RI: 1.45 | 2x | 0.81µm x 0.81µm | 1.62µm | 2 | Dorsal 1313µm; Sag. 2198µm | 2 (multiview combined) | 25GB | 1h 15min |
| | | | | | | | | | | | |
| | | | | | | | | | | | |
| **Supp. Figure** | **Animal** | **System** | **Objective** | **Postmagnification** | **Image pixel size** | **z-step** | **Number of channels** | **Imaging depth** | **Number of merged stacks** | **Data volume** | **Imaging Time** |
| 1b | adult *Drosophila* | Confocal | Leica 20x Gly | | 1.250µm x 1.250µm | 1µm | 2 | 74µm | | 100MB | 11min |
| | | | | | | | | | | | |
| 4a | adult *Drosophila* | UM | corr. 4x RI: 1.45 | | 1.642µm x 1.642µm | 1.642µm | 1 | 500µm-800µm | | n.a. | n.a. |
| | | | | | | | | | | | |
| 5a | adult *Drosophila* | UM | corr. 10x RI: 1.45 | 0.5x | 1.302µm x 1.302µm | 2.432µm | 2 | 1884µm | | 6GB | 45min |
| 5b | adult *Drosophila* | Confocal | Leica 20x Gly | | 0.757µm x 0.757µm | 2.01µm | 2 | 267µm | | 280MB | 11min |
| | | | | | | | | | | | |
| 7a | Larva | UM | corr. 10x RI: 1.45 | | 0.651µm x 0.651µm | 0.651µm | 2 | 895µm | 2 | 16GB | 1h20min |
| 7b | Larva | UM | corr. 10x RI: 1.45 | | 0.651µm x 0.651µm | 0.651µm | 2 | Clipped plane of 7a | 2 | n.a | n.a |
| | | | | | | | | | | | |
| 8a | Pupa | UM | corr. 10x RI: 1.45 | 0.5x | 1.302µm x 1.302µm | 0.651µm | 2 | 801µm | 2 | 12GB | 60min |
| 8b | Pupa | UM | Olympus 25x RI:1.45 | 2x | 0.129µm x 0.129µm | 0.4µm | 1 | clipping plane of stack of 422µm | | 6GB | 30min |
| 8c | Pupa | Confocal | Leica 40x Oil | | 0.378µm x 0.378µm | 1.09µm | 2 | one image | | 1MB | <1min |
| 8d | Pupa | Confocal | Leica 40x Oil | 1.8x Zoom | 0.215µm x 0.215µm | 0.59µm | 2 | 44µm | | 160MB | 6min |
| 8e | Pupa | Confocal | Leica 63x Gly | 1.8x Zoom | 0.135µm x 0.135µm | 0.04µm | 1 | one image | | 1MB | <1min |
| 8f | Pupa | Confocal | Leica 63x Gly | 1.8x Zoom | 0.137µm x 0.137µm | 0.71µm | 1 | 52µm | | 80MB | 6min |
| | | | | | | | | | | | |
| 9a | adult *Drosophila* | UM | corr. 10x RI: 1.45 | 0.5x | 1.302µm x 1.302µm | 2.916µm | 2 | 2145µm | | 6GB | 50min |
| 9b | adult *Drosophila* | UM | corr. 4x RI: 1.45 | 2x | 0.81µm x 0.81µm | 1.62µm | 1 | 1061µm | | 800MB | 12min |

| | | | | | | | | | | | |
|---|---|---|---|---|---|---|---|---|---|---|---|
| | | | | | | | | | | | |
| 10a | adult *Drosophila* | Confocal | Leica 20x Gly | | 0.757µm x 0.757µm | 2µm | 2 | 136µm | 2 | 288MB | 25min |

UM = ultramicroscope, corr. =corrected

**Supplementary Table 3 |** Video specifications

| Supp. Video | Animal | System | Objective | Postmagnification | Image pixel size | z-step | Imaging direction | Imaging depth |
|---|---|---|---|---|---|---|---|---|
| 1 | Larva | UM | corr. 10x RI: 1.45 | | 0.651µm x 0.651µm | 0.651µm | Dorsal | 895µm |
| | | | | | | | | |
| 2 | Prepupa | UM | corr. 10x RI: 1.45 | | 0.651µm x 0.651µm | 0.651µm | Dorsal | 936µm |
| | | | | | | | | |
| 3 | adult *Drosophila* | UM | corr. 10x RI: 1.45 | 0.5x | 1.302µm x 1.302µm | 2.916µm | Dorsal | 2145µm |
| | | | | | | | | |
| 4 (Multiview) | adult *Drosophila* | UM | Olympus 25x RI:1.45 | | 0.259µm x 0.259µm | 0.259µm | Coronal | 196µm |
| | adult *Drosophila* | UM | Olympus 25x RI:1.45 | | 0.259µm x 0.259µm | 0.259µm | Sagittal | 318µm |
| | | | | | | | | |
| 5 | Pupa | UM | Olympus 25x RI:1.45 | | 0.259µm x 0.259µm | 0.259µm | Dorsal | 512µm |
| | | | | | | | | |
| 6 | adult *Drosophila* | UM | corr. 10x RI: 1.45 | 2x | 0.324µm x 0.324µm | 1.539µm | Sagittal | 672µm |
| | | | | | | | | |
| 7 | adult *Drosophila* | UM | corr. 10x RI: 1.45 | | 0.651µm x 0.651µm | 0.651µm | Sagital | 601µm |
| | | | | | | | | |
| 8 (Multiview) | adult *Drosophila* | UM | corr. 4x RI: 1.45 | 2x | 0.81µm x 0.81µm | 1.62µm | Dorsal | 1313µm |
| | adult *Drosophila* | UM | corr. 4x RI: 1.45 | 2x | 0.81µm x 0.81µm | 1.62µm | Sagital | 2198µm |

UM = ultramicroscope, corr. = corrected
