# Supplementary material for: High-resolution ultramicroscopy of the developing and adult nervous system in optically cleared Drosophila melanogaster
Source: Nat Commun. 2018 Nov 9;9:4731. doi: 10.1038/s41467-018-07192-z (PMC6226481; doi:10.1038/s41467-018-07192-z)
Supplement: Supplementary file 12 — Description of Additional Supplementary Files [file 41467_2018_7192_MOESM12_ESM.pdf]

Description of Additional Supplementary Files

File Name: Supplementary Movie 1
Description: GFP signal in 3rd instar larve showing trachea, mid gut and salivary glands.

File Name: Supplementary Movie 2
Description: GFP signal in prepupa showing projection of photoreceptors in the developing visual system and the innervation of the segmental nerves into the ventral nerve cord.

File Name: Supplementary Movie 3
Description: GFP signal in appendages of adult fly.

File Name: Supplementary Movie 4
Description: Multi-view combined stack of GFP labelled dorsal cluster neurons in homozygous mutants of the neuronal cell adhesion molecule Neuroglian showing the specific loss of the commissural tract in adult Drosophila.

File Name: Supplementary Movie 5
Description: GFP signal in pupa showing visual system and olfactory system.

File Name: Supplementary Movie 6
Description: GFP signal in the undissected projection of sensory neurons from their receptors in eyes, antennae, maxillary palps and labellum to their respective central processing areas in the brain of adult fly.

File Name: Supplementary Movie 7
Description: GFP signal in the projections of the antennal nerve to the antennal lobe of adult fly.

File Name: Supplementary Movie 8
Description: Multi-view combined stack of Dscam-GFP signal in the whole adult fly.
